# Safety and efficacy of intravenous thrombolysis for acute ischemic stroke secondary to intracranial vertebrobasilar artery dissection

**DOI:** 10.3389/fneur.2025.1528168

**Published:** 2025-04-30

**Authors:** Nuo Wang, Wei Liu, Huangbin Lin, Benqiang Deng, Kaijun Zhao, Tao Wu

**Affiliations:** ^1^Department of Neurology, The Fourth People's Hospital Affiliated to Tongji University, Shanghai, China; ^2^Department of Neurology, Center of Cerebrovascular Disorders, Changhai Hospital, Second Military Medical University, Shanghai, China; ^3^Department of Neurosurgery, Shanghai East Hospital, School of Medicine, Tongji University, Shanghai, China

**Keywords:** acute ischemic stroke, vertebrobasilar artery dissection, intravenous thrombolysis, HR-MRI, safety and efficacy

## Abstract

**Objective:**

The safety and effectiveness of thrombolysis in patients with intracranial artery dissection (IAD) are still controversial. This study aims to assess the safety and efficacy of intravenous thrombolysis (IVT) in patients with intracranial vertebrobasilar artery dissection (i-VBAD) related acute ischemic stroke (AIS).

**Methods:**

A retrospective review of 32 patients admitted to our Neurovascular Center between January 2016 and June 2021 with AIS due to i-VBAD was conducted. Patients were identified and divided into IVT group (*n* = 8) and non-IVT group (*n* = 24) receiving standard antithrombotic therapy.

**Results:**

The mean age of the 32 patients was 49.28 ± 15.6 years, with a male predominance (87.5%). All patients presented with clinical manifestations consistent with posterior circulation infarct. Patients in the IVT group were significantly older than those in non-IVT group (58.88 vs. 46.08 years, *p* = 0.043) and had a higher prevalence of diabetes mellitus (50.0% vs. 8.3%, *p* = 0.023). No intracranial hemorrhage was observed in of the eight patients in IVT group. An excellent functional outcome, defined as an modified Rankin Scale score of 0–1, was achieved in all eight patients in the IVT group (100%) compared to 15 of the 24 patients in the non-IVT group (62.5%, *p* = 0.070). Although the difference did not reach statistical significance, the trend suggested a potential benefit of IVT in this patient population.

**Conclusion:**

IVT appears safe with no hemorrhagic complications in i-VBAD patients. It may offer better functional outcomes compared to standard therapy. Larger, prospective, multicenter studies are needed for definitive validation.

## Introduction

Stroke is a leading cause of disability and death in China (1), with a tendency to affect younger populations. Cerebral vascular dissection is a significant cause of stroke in young people, accounting for approximately 10–25% of cases (2). In Asian populations, the incidence of intracranial artery dissection (IAD) is higher than that of extracranial artery dissection (EAD) (3, 4), with the most common type of IAD being intracranial vertebrobasilar artery dissection (i-VBAD) (5, 6). Unruptured i-VBAD can lead to severe and potentially fatal ischemic events. However, as previous long-term follow-up studies have shown no occurrence of subarachnoid hemorrhage (SAH) in patients with unruptured i-VBAD (3, 7), it is generally accepted that treatment for unruptured i-VBAD should focus on managing ischemic stroke rather than preventing bleeding.

Two international multicenter studies have demonstrated that IVT treatment of acute ischemic stroke (AIS) within 4.5 h after symptom onset is both safe and effective (8, 9). Most randomized controlled trials investigating the efficacy of IVT did not specifically exclude patients with AIS caused by IAD. However, the safety and effectiveness of IVT in patients with IAD remain controversial. In fact, IVT was also not recommended for these patients by 6 out of 9 experts in the 2021 edition of European Stroke Organization (ESO) guidelines on IVT for AIS (10). Additionally, an increasing number of hospitals emergency rooms are utilizing multimodality imaging to evaluate brain vessels in suspected stroke patients. This comprehensive imaging can make it challenging for physicians to decide whether to administer thrombolysis to AIS patients within the treatment time window if i-VBAD is detected. In this study, we present the detailed processes and outcomes of IVT and non-IVT treatments in patients with AIS caused by i-VBAD, with the hope to help clinicians deal with similar cases more effectively.

## Patients and methods

All VBAD patients admitted to our hospital between January 2016 and June 2021 were reviewed retrospectively. Patients with extracranial vertebral artery dissection, asymptomatic vertebrobasilar dissection, and i-VBAD presenting as SAH were excluded from the study. Written informed consent was obtained from the patients or their legal representatives, and the study protocol was approved by the Ethics Committee of our Hospital. No vulnerable patients were included in this study. The study was conducted in accordance with the provisions of the Declaration of Helsinki, and this retrospective study did not cause any harm to the patients. The aim of the present study was to investigate the safety and effectiveness of IVT alone in i-VBAD patients in clinical practice. Patients who received endovascular therapy during the acute phase of stroke or within 3 months were excluded from the study. In our series, i-VBAD was diagnosed through a combination of multimodal CT scans and MRI, ensuring a comprehensive assessment for all patients. The diagnosis was established based on several criteria (11–13): the presence of clinical symptoms consistent with posterior circulation ischemia, such as dizziness, ataxia, and dysphagia; visualization of an intramural hematoma on cranial MRI, which is the gold standard for dissection diagnosis; detection of abnormal perfusion in the posterior circulation or occipital lobe via CT perfusion imaging; and characteristic findings on cerebral CT angiography (CTA), including a beaded appearance or tapering of the vertebral artery, indicating potential dissection.

Prior to IVT, a multimodal CT scan, including non-contrast CT brain imaging, CT perfusion, and cerebral CT CTA, was performed. The decision to administer IVT was based on several key factors (9): adherence to the 4.5-h therapeutic time window from symptom onset to treatment initiation, the presence of sudden neurological deficits, the results of multimodal CT imaging to exclude significant intracranial hemorrhage, and the absence of any contraindications. Patients received alteplase at a dose of 0.9 mg per kilogram of recent body weight, with 10% administered as a bolus, followed by micro-pump delivery of the remaining 90% as a constant infusion over a period of 60 min (8). Eight patients showed no signs of intracerebral hemorrhage 24 h after thrombolysis, and were subsequently administered antithrombotic therapy.

Brain MRI was performed in all patients. MRI examination included T1-weighted (T1WI), T2-weighted (T2WI), fluid attenuated inversion recovery sequence imaging and DWI. Additionally, partial patients underwent high-resolution MRI (HR-MRI) to assess for the presence of vessel dissection when necessary. The clinical data of patients, including age, gender, vascular risk factors, the National Institute of Health stroke scale (NIHSS) score, pre-hospital time, and 90-day modified Rankin Scale (mRS) score, were collected. Pre-hospital time was defined as the interval from symptom onset to the documented time of hospital arrival.

Statistical analyses were performed with SPSS 21.0 (IBM SPSS Statistics for Windows, IBM Corp., Armonk, NY). Continuous variables were summarized as mean (standard deviation, SD) or median (interquartile range, IQR). Categorical variables were presented as percentages. Categorical variables were analyzed and compared using Fisher’s exact test, and continuous variables were compared by using the independent samples *t*-test. NIHSS score and pre-hospital time that did not fit a normal distribution were compared by Mann-Whiney *U* test.

## Results

### Clinical features

**Figure 1:**
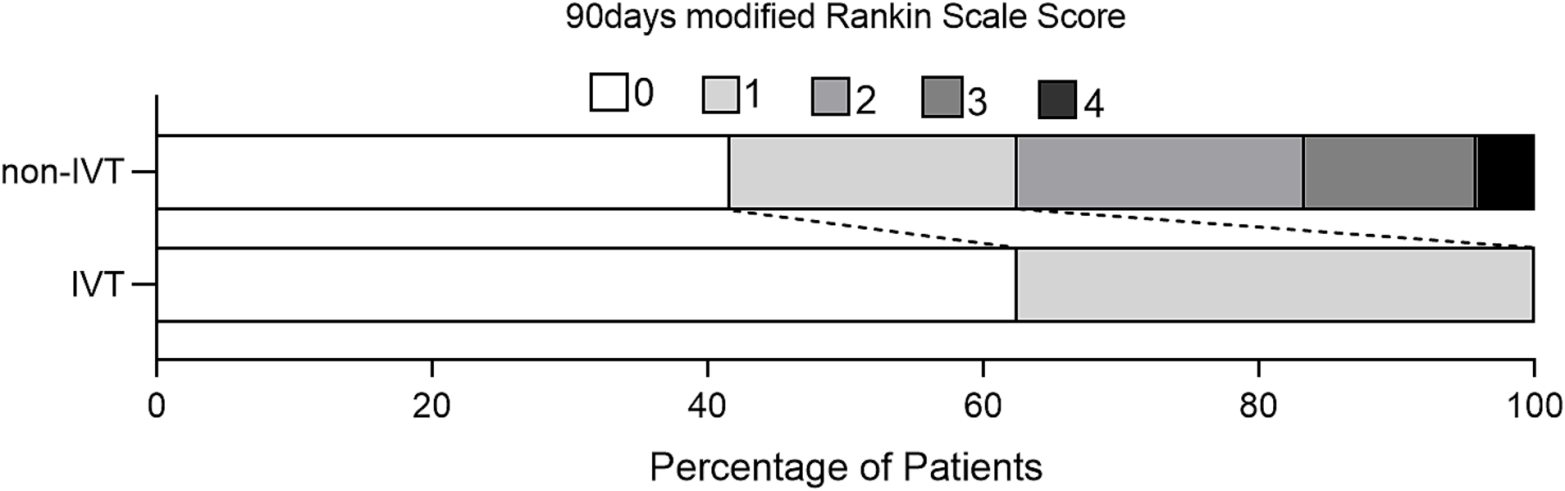
Distribution of scores on the modified Rankin Scale at 90 days for patients without and with IVT.

The mean age of the 32 patients was 49.28 ± 15.6 years, with a male predominance (*n* = 28). Demographics, risk factors, and outcomes are summarized in Table 1. Patients in the IVT group were older than those in the non-IVT group (58.88 vs. 46.08 years, *p* = 0.043) and had a higher incidence of diabetes (50.0 vs. 8.3%, *p* = 0.023). There was no significant difference in the distribution of gender, hypertension, current smoker, migraine history, and NIHSS score between the two groups (*p* > 0.05). The pre-hospital time in the IVT group was shorter than that in the non-IVT group (1.75 vs. 5.5 h, *p* = 0.007). Excellent outcome (mRS ≤ 1) at 90-day follow-up were achieved in all eight patients in the IVT group and in 15 patients (62.5%) in the non-IVT group (Figure 1). The lack of a significant difference in outcomes between the two groups (*p* = 0.07) may be attributed to the small sample size.

**Table 1:** Demographics, risk factors, clinical history, and outcomes of patients with i-VBAD.

Characteristics	Total (*n* = 32)	IVT group (*n* = 8)	non-IVT group (*n* = 24)	*p* value
Age, mean (SD), year	49.28 (15.6)	58.88 (15.2)	46.08 (14.7)	0.043
Male (%)	28 (87.5)	8 (100)	20 (83.3)	0.550
Hypertension (%)	15 (46.9)	6 (75.0)	9 (37.5)	0.106
DM (%)	6 (18.8)	4 (50.0)	2 (8.3)	0.023
Current smoker (%)	17 (53.1)	5 (62.5)	12 (50.0)	0.691
Migraine history (%)	2 (6.3)	1 (12.5)	1 (4.2)	0.444
NIHSS score (IQR)	4 (2.5)	4 (3.4)	3.5 (2.5)	0.848
Pre-hospital time (IQR), h	3 (1.6, 14)	1.75 (0.85, 2)	5.5 (2, 24)	0.007
Neck or head pain (%)	16 (50)	2 (25.0)	14 (58.3)	0.220
Excellent outcome (%)	23 (71.9)	8 (100.0)	15 (62.5)	0.070

DM, diabetes mellitus; NIHSS score, National Institute of Health stroke scale score; h, hour.

**Figure 2:**
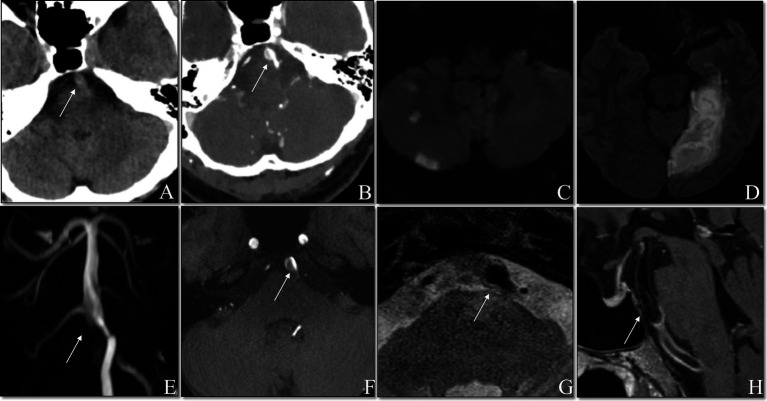
Non-contrasted cranial CT showed suspicious double lumen and intimal flap **(A)**. Contrasted cranial CT showed the mural hematoma **(B)**. DWI showed fresh posterior circulation infracts **(C,D)**. TOF MRA demonstrated the basilar artery with a dissecting aneurysm **(E)**. TOF sources showed the mural hematoma **(F)**. Axial T2WI showed the dilated lumen of basilar artery **(G)**. Contrast-enhanced T1WI showed an enhancement along the surface of the mural hematoma **(H)**.

Patient 5 discontinued IVT at 23 min (total dose of alteplase: 24.5 mg) due to the complaint of a headache, although the dysarthria improved and repeat MRA showed no enlargement of the intramural hematoma and no expansion of the dissection-flap. Patient 4 discontinued thrombolysis after a 6 mg bolus of alteplase due to fear of serious hemorrhage as subsequent brain CTA revealed a dissecting aneurysm proximal to the basilar artery (Figure 2). Patient 6 lost consciousness at 20 min after thrombolysis, and no bleeding was found in the head CT. Thrombolysis was continued, and the patient regained consciousness 15 min later. The symptoms in Patient 1 were significantly aggravated 55 min after IVT, but head CT showed no bleeding, and therefore IVT continued until the end, with the significant relief of dizziness and dysphagia. The thrombolytic process was successful, and symptoms improved after IVT in the remaining four patients (shown in Table 2).

**Table 2:** Summary of clinical features of patients treated with IVT.

Patients	Sex/Age (years)	NIHSS score	Site of dissection	The process of IVT	IL on MRI	90d mRS	Recanalization
1	M/42	4	LV4	Symptoms aggravated	Left medulla	0	No
2	M/67	4	LV4	Stable	No	1	No
3	M/58	3	LV4	Stable	No	0	No
4	M/41	4	LV3, LV4, BA	Stop after bolus injection	Posterior circulation	0	No
5	M/38	5	LV4	Headache occurring during IVT	No	0	No
6	M/75	2	RV3, RV4	Transient loss of consciousness during IVT	Posterior circulation	0	Yes
7	M/42	5	RV4	Stable	No	1	No
8	M/75	3	LV4, RV4	Stable	Left cerebellum	1	Null

NIHSS score, National Institute of Health stroke scale score; mRS, modified Rankin scale; IL, infarct location; Null, the blood vessels were not reviewed.

### Neuroimaging results

In the IVT group, V4 dissection was observed in seven patients, and one patient had combined V4 and basilar artery (BA) dissection. In the non-IVT group, V4 dissection was present in 22 patients, BA dissection in one patient, and V4 combined with bilateral V1 dissection in one patient.

Of the eight patients in the IVT group, one had cerebellar infarction, two had brainstem infarction, two had multi-territory areas of infarction, and three had no fresh infarction. Of the 24 patients in non-IVT group, 13 had brainstem infarction, three had cerebellar infarction, seven had multi-territory areas of infarction, and one had thalamic infarction on MRI-DWI. No bleeding was observed in any of the patients in the IVT group, as confirmed by multiple head CT examinations.

Three months later, brain vessel examinations showed no vascular change in five patients (83.3%) in the IVT group compared to eight patients (44.4%) in the non-IVT group. Recanalization was observed in one patient (16.7%) in the IVT group and 10 patients (55.6%) in the non-IVT group. Two patients in the IVT group and 6 patients in the non-IVT group did not undergo follow-up brain vessel examinations.

## Discussion

Arterial dissections are caused by a tear in the intima or media of the vessel wall, resulting in bleeding within the arterial wall and leading to intramural hematoma formation (6, 14, 15). Patients with IAD may manifest as SAH, AIS or local compression symptoms, with acute cerebral infarction being the most common form (3, 16). In our series, symptoms were relieved following aggravation during or after IVT, and all eight patients in the IVT group recovered well, indicating that thrombolytic therapy for AIS caused by i-VBAD was effective. Subsequent imaging examinations, including CT and/or MRI, confirmed no hemorrhage, indicating that thrombolytic therapy is also safe for i-VBAD patients. However, as symptom fluctuation may cause distress to the patient, adequate communication prior to IVT is essential.

Arterial dissection is increasingly recognized as a cause of stroke due to growing familiarity with its clinical features and advancements in neurovascular imaging. In our study, patients in the IVT group were older and had a higher proportion of diabetes, suggesting that clinicians might be hesitant to diagnose AIS in younger individuals with posterior circulation ischemia who lack vascular risk factors. Emergency multimodal imaging is valuable for diagnosing VBAD-related cerebral infarction, and our findings may assist clinicians in managing similar cases.

Although many studies have demonstrated the safety of thrombolysis for patients with EAD (17, 18), the risk of intracranial hemorrhage can be higher in IAD, especially when the lesion is located in the posterior circulation (15, 19). Thrombolysis may increase the risk of intramural hematoma enlargement, dissection flap expansion, and bleeding (19, 20). Consequently, European stroke experts generally do not recommend IVT for patients with IAD (10). Moreover, some authors believe that thrombolysis could lyse the thrombus within the arterial wall, which may potentially increase the risk of dissection expansion due to the added shear force on the damaged vessel wall (21). The media layer, which is crucial for vessel strength, is thinner in intracranial arteries (22, 23). Unlike the local compression symptoms caused by the rupture of an EAD, a rupture of an IAD can result in SAH, which is more dangerous. These factors may also contribute to why clinicians are reluctant to consider thrombolysis for patients with IAD. There is limited literature about the treatment of IAD with IVT. Some individual studies report the risk of symptomatic intracranial hemorrhage (sICH) and other serious adverse events of thrombolysis was not increased in IAD patients (24, 25). It is well-known that trauma, mechanical stress, or ever sudden neck movements or stretching is a common mechanisms causing arterial tears (14). Arterial dissections almost always occur in regions where arteries are mobile and not anchored to bony structures or other arteries (6, 26). In contrast, intracranial arteries are fixed to the brain’s surface, making them less susceptible to external forces. Therefore, the risk of hematoma enlargement and dissection rupture caused by thrombolytics alone may primarily be a theoretical concern.

In our study, no instances of ICH following IVT were observed in patients with i-VBAD. Tsivgoulis et al. reported a relatively low incidence of sICH in similar populations, with an sICH rate of 2.5% in a multicenter study involving 122 patients with dissection-related ischemic stroke treated with IVT (25). These findings suggest a favorable safety profile for thrombolysis in such cases. Similarly, Bernardo et al. found no sICH in a single-center series of 15 patients (27). Additionally, a meta-analysis by Vergouwen et al. reported a pooled sICH rate of 3.3% for cervical artery dissection, further supporting the notion that thrombolysis may not significantly elevate the risk of hemorrhagic complications in patients with arterial dissection (28). The CADISS trial highlights the diagnostic complexities inherent to dissection-related strokes, indicating variability in patient outcomes (29).

Most patients with i-VBAD in our series recovered well by the 90-day follow-up, which aligns with previous findings that those with ischemic presentations often had favorable outcomes (3, 7). Lower initial NIHSS scores and posterior circulation lesions might contribute to these positive outcomes (25). Dual antiplatelet therapy (DAPT) is a viable treatment option for patients with minor ischemic stroke (NIHSS < 3–5 points) and has been shown to be at least as effective as single antiplatelet therapy in preventing recurrent stroke (30–32). This is particularly relevant for patients with i-VBAD without distal vessel occlusion. Dissection can lead to ischemic stroke either through thromboembolism or, less commonly, from hemodynamic insufficiency due to severe arterial stenosis or occlusion (33, 34). Since the propagation and embolization of red (erythrocyte–fibrin) thrombi is the main mechanism (6), Professor Caplan suggested that thrombolysis could be an ideal treatment for patients with IAD, provided it is administered within the appropriate time window and without large area infarction. Symptom fluctuations during thrombolytic treatment may be attributed to the thrombolysis procedures.

The optimal secondary stroke prevention regimen for patients with IAD remains unclear. Patients in the IVT group were treated with IVT alone and then started on antithrombotic therapy, considering that imaging studies show spontaneous recanalization in 20–58% of patients with IAD (35). Ischemic lesions are generally managed with medical therapy. Endovascular therapy should only be considered if the patient experiences recurrent strokes despite medical treatment (36).

A retrospective study of unruptured spontaneous intracranial VAD suggested that female gender, the nonsmoking status, and the absence of posteroinferior cerebellar artery involvement might be associated with spontaneous vascular normalization (7). The proportions of male and current smokers were similar between the two groups; however, the proportion of spontaneous recanalization in the non-IVT group is consistent with previous reports and is higher than that in IVT group. Whether alteplase adversely affects the healing of artery dissection requires further investigation. Quicker treatment improves AIS outcomes by restoring blood flow sooner and reducing ischemia duration, possibly explaining the better outcomes in the IVT group. But further studies are needed to confirm this. Furthermore, the absence of randomization and the small sample size, which may introduce biases and reduce statistical power. It’s important to note that the small number of patients from a single institution may not be representative. Prospective, multicenter studies of patients with AIS secondary to IAD are needed to determine the optimal treatment regimen.

## Conclusion

In conclusion, this preliminary study suggests that IVT may be safe and effective for AIS caused by i-VBAD, with no observed hemorrhage. However, the small sample size and lack of randomization limit the strength of this conclusion. The trend toward better outcomes at 90 days should be interpreted with caution. Larger, prospective, multicenter studies are needed to confirm the safety and efficacy of IVT in i-VBAD patients.

## Data Availability

The original contributions presented in the study are included in the article/supplementary material, further inquiries can be directed to the corresponding authors.
